# Traite des Maladies Ventenses, ou Lettres sur les Causes et les Effets de la Presence des Gaz ou Vents dans les Voies Gastriques, et sur les Moyens de Guerin ou de Soulager ces Maladies

**Published:** 1841-08

**Authors:** 


### REVIEWS.

*Traite des Maladies Ventenses, ou Lettres sur les Causes et les Effets de la Presence des Gaz ou vents dans les voies Gastriques, et sur les Moyens de Guerin ou de Soulager ces Maladies ; par* M. P. Baumes, *Chirurgien en chef de l'Hospice de L'Antiquaille de Lyon, &c. &c.*

A Treatise on Gaseous Diseases, or Letters upon the Causes and Effects of the presence of gases or wind in the Alimentary Canal, and upon the means of curing or relieving these diseases; by M. P. Baumes. Small 8vo. pp. 223. Paris, Lyons, and Montpelier. 1837.

The formation of gases or wind in the human body, regarded either as a physiological or pathological phenomenon, has attracted but little attention from physicians, foreign or American. In our standard works of physiology the function of gasification is not considered, and in the most popular Treatise of Pathology and Morbid Anatomy of this day, (Andral) the whole subject is despatched in a very few lines, in a manner altogether unsatisfactory.

The monograph before us supplies, therefore, a want which we doubt not has often been felt; and as the author was himself the subject of a very troublesome gaseous affection, and as he has devoted much of his time to the investigation of the subject, in a manner truly practical and experimental, many new and valuable facts have been given to the profession as the result of his labors.

We propose giving to the reader the leading facts and principles, laid down in this treatise, touching the pathology and treatment of these affections. The work consists of twelve letters in which the author considers successively, in a plain and easy style, the various topics embraced in the investigation. After giving an historical summary of all preceding works on the subject, (the substance of which is, that, altho' Hippocrates had said something of gases, although Van Helmont had touched upon them, and, of late, although some few distinguished pathologists had made gasification a subject of study, little or nothing has been said or done to afford any positive knowledge of the matter,) the author proceeds to lay down the various propositions and conclusions which the work contains.

"The gases in the alimentary canal arise from different causes. Sometimes it is the atmospheric air which has entered by the movements of deglutition—of respiration, or the atmospheric air which other gases contain always in greater or less quantity in the food. At other times they are the result of an operation of a chemico-vital nature, like that which constitutes digestion; but above all, whilst there is imperfect digestion, or complete indigestion. They seem to arise again by general belief, at the commencement of decomposition of some materials which have remained for too long a time in the intestinal canal; but you will know better hereafter what estimate to place upon this opinion. The kind of putrefaction—of decomposition, which gangrene exhibits, like one sees in a case of strangulated hernia, occasions, in the same way, their generation."

"With the exception of these cases, their production is owing to a *vital action*, a genuine exhalation which may occur in a state of health, but which more frequently is the result of a state of excitement—irritation of the alimentary canal, and oftentimes, to appearance, of atony. In the same manner as a bloody, mucous, serous, or purulent discharge exists, so does a gaseous discharge exist; and the development of gas constitutes one of those drains by which nature reacts against divers excitements, in obedience to the principle established by Hippocrates, "*ubi stimulus, ibi fluxus.*"

As resolution, suppuration, ulceration, &c. are regarded as terminations of inflammation, so also may the production of gas; which phenomenon may be designated by the term *flatusation.* It is regarded by our author as a favorable symptom when inflammation is accompanied with abundant gaseous products, inasmuch as they are a method of disengorgement, "a favorable way which prevents Nature from committing herself to other morbid works more or less dangerous—a way which one might be happy to obtain, but which is as difficult to obtain as a hemorrhage by the exhalents." On the contrary, when, during the existence of inflammation, there has been an abundant development of gas, and then a sudden suppression, every danger is to be apprehended. We know the danger arising from the suppression of an habitual discharge—of an eruption upon the skin—of a copious perspiration during fever—of the discharge from a blistered surface, while yet there is intense inflammatory action going on in the internal organ on which the blister has been applied. Now in inflammation of any hollow organ, or an organ having an excretory duct, if there seems to be a termination by flatusation, and while this favorable discharge is going on, a sudden suppression should occur, as a matter of course, the inflammation would become more intense; and it becomes the physician here to direct his remedies in such a manner as to reestablish the gaseous discharge.

The phenomena of the formation and disappearance of gases, therefore, become important means of diagnosis and prognosis.

"The effect of gases in the alimentary canal are extremely varied. They often frighten by the serious symptoms they afford, and have more than once led experienced practitioners into error. Sometimes, without finding issue by the mouth or anus, they are absorbed rapidly, or more or less slowly; sometimes by the lymphatic system and disappear with the lymph—then again by the venous system, * * * * * and they seem, while combined with the blood, to resume their elastic form—to dilate and compress organs, to clog and arrest the circulation, to produce formidable accidents in the heart and brain, and death itself."

Thus much for a brief summary of the causes and effects of gaseous discharges. The author next adverts, in the same brief manner, to the treatment.

"The treatment of gaseous discharges in the alimentary canal may be *vital*, *chemical*, or *mechanical*: VITAL, when one desires to modify the organism—the same vital action which generated the gases; or to effect their expulsion by acting upon the contractility of the gastro-intestinal tube: CHEMICAL, when substances are introduced into the alimentary canal which operate by combining themselves with the gases, absorbing them, or causing them in part to disappear: MECHANICAL, when one wishes to extract them either by the mouth or, particularly, by the anus, or even by any part of the abdominal parietes by means of a surgical operation."

After this brief summary, the author discusses, at length, the three topics which have been glanced at, viz. the *causes*, *effects*, and *treatment* of gaseous discharges.

I. *Causes of the presence of gas or wind in the alimentary canal.*

The author alludes to the fact that infants have been born with a large quantity of gas in the alimentary canal, and shows that they cannot be attributed to any thing else than to a particular condition of the mucous membrane, for the idea of decomposition is out of the question.

Gaseous exhalation is a physiological, or pathological phenomenon.

1st. "With regard to gaseous exhalation in a natural state among plants, as well as among animals, the facts are numerous and well known every where. Who is ignorant of the phenomena of gaseous exhalation and absorption of leaves and of other parts of vegetables? And as to animals, does not the phenomenon of the swimming bladder of fishes, which forms itself during the act of respiration, and the exercise of the functions of the cutaneous system, render this exhalation evident? As to the digestive tube, in a state of health, particularly in certain persons endowed with a particular disposition, there is in it also, regularly, without doubt, exhalation and absorption of gas. If by a convenient opening made in the belly of an animal, one can draw out a small portion of the intestinal tube not containing any matter, then include it between two ligatures, the intestine thus tied, if allowed to remain in the abdomen, in a short time will, in the portion included between the ligatures, be distended with gas, while other parts of the mucous membrane will appear perfectly healthy."

"The phenomenon of gaseous exhalation in a state of health in the alimentary canal, has been very well noticed by Bernard Gaspard, (Dissertation, 1812.) This author has displayed a great deal of sagacity in the following passage, which seems to support strongly my method of considering gases in a morbid state:

"'It is said that a quantity of gas disengages itself constantly in the intestinal canal of man and animals, particularly during digestion. This gaseous exhalation occurs in persons of good health, but especially in those who are nervous, bilious, melancholic, and naturally windy; also in old age, in nervous, hypochondriac, hysterical, and chlorotic affections, &c.; or it creates the borborygmi and the rumbling noises incident to diseases. These gases are not fœtid; they are particularly azotic, going off by the anus with explosion; or others do not discharge themselves, and are, without doubt, re-absorbed. It seems, also, that they are especially exhaled in great quantity by a kind of option, at the time of the disappearance of emphysema, or during a lesion of the functions of the skin, of the cooling of the feet, &c. But the fact which best establishes the real vital exhalation of these flatuosities is, that they are susceptible of being modified in their quantity, their odour and nature, by divers cutaneous and pulmonary absorptions. Thus the winds which are produced in dissecting a cadaver are cadaveric, according to Bichat; those which are produced by breathing the odour of pus, or in remaining near large ulcers, are like purulent matter. The rubbing of sulphur seems to create a sulphurous odour.'"

As a pathological phenomenon, the author adduces several reasons to sustain the position that gaseous exhalation—or rather gastro-intestinal gases, result from a "state of excitation, irritation, or inflammation itself."

The following argument appears to be as satisfactory as any which he has offered.

"That which proves that Nature proceeds in the exhalation of gases as in all other exhalations, and that they are so many modes of action, so many different products of the same morbid phenomenon is, that all these exhalations often alternate one with another, succeeding each other, replacing one another, so that the augmentation or appearance of one induces the diminution or disappearance of another. A number of facts prove this." * * * * "The gaseous exhalation which supplies the place of other exhalations or secretions may occur in other organs at the same time that it exists in the alimentary canal. The diminution or suppression of urine, of sweat, of fœces, of the menses, of the lochia, of leucorrhea, of habitual epistaxis, of ptyalism, etc., have given rise to the development of gas in the alimentary canal, in the other cavities lined with mucous or serous membranes, in the cellular tissue, &c. &c."

The following cases among many others recorded, serve to illustrate the position taken by the author.

"M. Pau, a wine merchant, who resided at Guillotiere, came one day with his wife to consult me. The night previous they had suffered, the husband, with a considerable diarrhœa, nausea, excessive pains and cramps: the wife, with a violent colic accompanied with an eruption, above and below, of a prodigious quantity of wind, although she had never before been flatulent. All these symptoms ceased in the morning; but the same thing happened to them five days afterwards, and in the interval they were perfectly well. It was evident that the same cause, or a similar cause, appeared to have acted the second time, in the days of interval, to produce the same morbid phenomena. I convinced myself, by questioning them, that it could not be charged to an error in regimen, nor to the quality of food or drinks, nor to a strong moral emotion, nor fatigue, nor, in a word, to any one of the causes which more ordinarily produce similar phenomena; but I understood that the two evenings which preceded the two nights of sickness, they had gone to make up a party of pleasure at the room of a painter, where a strong odour of varnish was diffused at the time of their visit. I directed their attention to this circumstance, and they immediately remembered that when leaving the painter's they commenced to feel nausea, dryness of the mouth, and a general malaise. It seemed easy to me to understand from them that the true cause of their complaint was the vapour of varnish acting upon the alimentary canal. Having ceased visiting the painter's room the accident did not recur. Here it is evident that the same irritating cause produced, (according to the particular disposition of each individual,) in the digestive tube of one a mucous or sero-mucous discharge, and in the other a discharge entirely gaseous."

The following fact is worthy of notice:

"When inflammation attacks the gastro-intestinal mucous membrane in an individual disposed to gaseous exhalation, it is by this exhalation that nature manifests the commencement of the attack. *If inflammation acquires, suddenly or gradually, a very high intensity, no gaseous exhalation will occur where it is more intense, but may continue in those parts of the mucous membrane less inflamed, or less irritated.*

This comports with the well established pathological law, that the secretory stage of inflammatory action in any tissue of the body, is less active than the preceding congestive stage. For example, the burning and throbbing of a boil is relieved in a great degree so soon as suppuration commences. Irritation of the bowels as well as inflammation diminish, and indeed often become entirely relieved upon the occurrence of diarrhœa. The dropsies are all considered referable to a subacute inflammatory state, etc. etc. Now the principle which obtains in the secretion and exhalation of serum, mucus or pus, obtains also in the secretion and exhalation of gas.

Such is the substance of the author's views concerning the causes of the presence of gas in the alimentary canal. We come now to his second head,

II. *The effects of the presence of gas or wind in the alimentary canal.*

Among these effects, the principal will be found in the following list.

1. Enormous dilatation of the digestive tube.
2. Violent colic.

3. Compression of neighboring organs, resulting in disordered function, etc.

4. Absorption of gases by the lacteals and venous radicles; their passage into the circulation, producing,

5. Accidents in the brain, heart, &c. apoplxy, syncope, etc.

General dilatation of the digestive tube constitutes one form of tympanitis; the other form results either from a gaseous secretion from the peritoneum, or the escape of air from the bowels, by a perforation, into the cavity of this membrane. We believe the first form of the disease is the more common.

The writer of this review reported a case in the Western Journal Med. and Phys. Sciences, Vol. No. in which, after several days continuance of inflammation of the bowels, during which time the patient partook of no nourishment, save gum water, tympanitis took place, and so enormous was the distension, and so ineffectual were all remedies addressed to the disease, that it was decided upon, as a last resort, to introduce the trocar. This was done, and a great amount of wind escaped, but the operation was unavailing. The patient died, and a post mortem examination disclosed what was anticipated—an obstruction (consisting of a scirrhous growth) above the sigmoid flexure of the colon, which prevented the egress of wind, and, in its turn, aggravated, by its detention in the bowels, the inflammation which, without doubt, produced it. Now if from any cause, such as the one we have mentioned, spasmodic constriction, pressure of other abdominal organs, an enlargement of a mesenteric gland, a tumour, or intussusception of the bowels themselves, gas should be prevented from passing off, dilatation will not only result, but the gas acting as foreign matter will serve to increase the inflammation, just as retained pus or mucus; so that in all cases of tympanitis it is important to know, if possible, if there be any obstacle to the exit of gas, in order that we may form a proper diagnosis.

But it not unfrequently happens that retained gas, instead of acting upon the mucous lining of the stomach and bowels, exerts itself, as do many foreign matters, upon the muscular coat. Hence *Colic.*

In the third place, we are to notice the compression exerted upon neighboring organs, and the consequent derangements of function.

"In distending the rectum they press upon the bladder, prevent its dilatation, and cause frequent discharges of urine; or if the pressure be exerted near the neck of this organ, the result, on the contrary, is a retention of urine."

Here is an apparently very simple cause of two forms of urinary disease, which sometimes become very alarming—a cause which, we believe, few are apt to think of—a cause which is apt to exist very often; and we doubt not but that many a patient has been subjected to torture by fomentations, diluents, astringents, &c. &c., when, perhaps, the introduction of a rectum bougie would put an end to his sufferings.

"In distending the colon, or small intestine, they are apt to rise up in the stomach and occasion vomiting. Accumulated in the entire length of the intestinal tube, they press upon the diaphragm and hinder the respiration."

The abdominal viscera may likewise be displaced, or even lacerated, and otherwise injured by the mechanical pressure of gas in the bowels. The bowels themselves are particularly liable to eruption when so distended. Cases illustrating these effects are given by the author.

Local accumulations of gas simulate sometimes other diseases, and physicians are liable to errors in diagnosis. They have been mistaken for wens, abscesses, and aneurisms, being circumscribed and quite hard. Hence the importance of examining with care all signs and symptoms.

Gaseous accumulations in the alimentary canal, particularly if accompanied by gaseous secretion in other parts of the body, render the body specifically lighter than water, and effectually prevent it from sinking in this fluid.

The following short case, quoted by the author from Morgagni, illustrates the absorption of gas:

"A fisherman of Venice, at the close of his fortieth year, having a hernia, and being subject to windy diseases of the abdomen, was taken suddenly in his boat, and died on the spot."

An examination of the body, made by Morgagni and Santorini, showed that the abdomen was swelled by air, which distended the stomach and intestines. These organs were very red, and inflamed, and presented, also, a gangrenous colour near the hernia. All the veins of the abdominal organs were filled with air, and there was not a single vein in the whole body which was not distended by a black and frothy blood. This circumstance was particularly witnessed in the cavity of the cranium, in the sinuses and vessels of the dura mater—of the pia mater, either at the base of the brain, or rest of the surface, or in the ventricles. * * * * * * In circulating with the blood, gases may give rise to distensions, temporary compression of various organs, obstructions in the circulation, excessive pain, palpitations, chills, vertigo, &c. &c. "They may also vitiate the blood and unfit it for nutrition."

Gases may resume their elastic form after having been absorbed and carried into the circulation. This may happen, either in the intestinal canal, or in the various cavities and organs of the body. In the former event, they may either be discharged by the anus, or give rise to the various lesions already noticed; in the latter, they may not only exert a deleterious influence by compression, but by acting as foreign matter, impair the sensibility of organs, interfering with the functions of nutrition and secretion, and possibly interrupting the latter function by a hidden process of vital chemistry, and lastly, by giving rise to the formation of morbid products.

After occupying four letters in further discussing the pathology of gaseous exhalation, and defining his views of irritation and inflammation, and the relation of these to the former phenomenon, together with some remarks upon diagnosis and prognosis, which, in substance, have already been stated, the author comes to consider,

III. The Treatment. This may be *Medical, Chemical,* or *Surgical.*

MEDICAL TREATMENT.

"1st. *Bloodletting.* When there is sanguineous plethora, experience shows that Nature chooses to unload herself of the superabundance of blood, which fatigues her, by discharges of every kind, and principally by a gaseous discharge in the digestive tube. It is this which appears often in women at a critical period, who become windy, and who were not so previously, and who cease to be so after a bleeding. If sanguineous plethora be general, the bleeding should be general. If the plethora is abdominal only, bleeding at the anus would be better, particularly if there be any hemorrhoidal disposition. The hemorrhoidal discharge, when it appears, causes the gaseous discharge to disappear."

In cases of local irritation, pain in a particular part of the abdomen, increased at the moment of desire to expel wind, &c., a few leeches may be applied immediately over the painful part, and their application renewed, from time to time, as circumstances may seem to require. Oily embrocations, fomentations, and cataplasms, are also useful.

"2nd. *Tonics.* When by a defective innervation, arising from an abuse of debilitating regimen, or the action of any debilitating cause whatever; when by a state of relaxation of the mucous tissue, there is a feeble and insufficient action of the digestive fluids in the process of digestion, tonics are proper. I advise the use of camomile tea, tea of bitter orange peel, gentian root, &c."

"3rd. *Stimulants.* These may be employed, when one wishes to impart a strong and immediate action to the muscular coat for the expulsion of gases. It is thus that the infusions of mint, coriander, canella, anise, sage, &c., various elixers, &c. prove useful; but in general these substances are injurious."

"4th. *Narcotics* may be very useful when, in persons of very irritable stomachs and bowels, the distention of one coat accompanies the constriction of another, as in cases of colic, cramp, &c. In these cases, I am accustomed to soothe the pain momentarily, without effecting the expulsion of any wind, with a quantity of syrup of poppies, dissolved in lettuce or orange water. * * * * * Embrocations with oil of morphine, or poppy; fomentations, well saturated with a decoction of henbane, poppy heads, &c., are useful in such cases. I am often successful with light friction, upon the parts which seem to correspond to a spasmodic constriction, with a pommade composed of equal parts of belladonna and Galieus cerate. The application of the leaves of belladonna produces, in some instances, the same result. It seems that this plant acts upon the constricted part of the intestine as it does upon the iris, or upon an orifice, the edges of which are spasmodically clasped."

"5th. *Diuretics and Diaphoretics* offer no other advantage than to direct the current of the circulation to important organs, which act as emunctories of the system, and possibly to substitute one discharge for another. But in general they are hurtful, because they are more or less stimulating to the intestines; moreover, the propriety of their administration demands, on the part of the physician, considerations which are applicable only to the particular case under notice."

"6th. *Emetics* are not admissible, except when the stomach contains a large quantity of food, or some indigestible aliment, or irritating substance, calculated to excite the mucous membrane to gaseous exhalation."

"7th. *Purgatives* are, in general, very injurious. I advise that windy persons should purge themselves as little as possible."

"8th. *Antispasmodics.* These remedies are useful when they destroy the great susceptibility, or irritability of the mucous membrane, or whenever they overcome the nervous erethism occasioned by the great distension of the mucous membrane, which the winds have occasioned by their elasticity and accumulation. Such are asafœtida, valerian, ether, musk, &c. &c."

"9th. *Topical Revulsives.* When the winds arise from inflammation, these topical medicines placed upon the limbs, but especially opposite the parts of the digestive tube diseased, produce, sometimes, excellent effect: such as sinapisms, vesicatories, &c. Cups also seem to be very useful in most cases."

### CHEMICAL TREATMENT.

The author expresses the opinion, founded upon observation, that no substance exists which is capable of absorbing the gases exhaled in the alimentary canal, without proving injurious itself as an irritant. Magnesia, which many suppose acts as an absorbent, he thinks, exerts its beneficial effect by virtue of its laxative power, converting the gaseous into a liquid discharge.

"A sound theory of the formation of gases, and the correct appreciation of the generality of facts, which practical medicine affords, seems, in general, to reject these means (absorbants) as never remedying the cause, and as never having any other than an inefficient action upon the effect."

### SURGICAL OR MECHANICAL TREATMENT.

"Winds may be drawn out of the alimentary canal by means of a canula or sound, to the extremity of which a syrynge or sucking pump is adapted. This procedure is easier in its application to the anus and rectum, than to the œsophagus and stomach. I have employed this means (in its applicution to the rectum) with success in several persons; and I have occasionally received some benefit by it myself. It is sufficient, sometimes, to introduce a gumelastic tube to the depth of several inches, in order to give vent to the wind, without any resort to pumping or sucking."

It is difficult, however, to arrive at gases in the small intestine, and not unfrequently spasmodic constrictions and feculent matter in the large intestine, prevent, almost altogether, the passage of gas, notwithstanding the use of a pump.

Paracentesis is proposed as a dernier resort.

"Finally, it is proposed, in these desperate cases, to puncture simultaneously, with a small trocar, the parietes of the abdomen and the walls of the intestinal tube at the point most distended."

A few remarks upon diet and regimen, drawn from Fodere, together with the repetition of what has been laid down as a palliative treatment, in cases requiring immediate relief, (the administration of sedatives and antispasmodics, and the external application of opiates and counter-irritants,) close the work.

We have thus endeavored to present a condensed abstract of this valuable little treatise. We know that we have profited by its perusal, and if the review here offered to our professional brethren, shall lead to the investigation of a much neglected part of pathology, our object will be fully accomplished.

